# The effect of 6 days of alpha glycerylphosphorylcholine on isometric strength

**DOI:** 10.1186/s12970-015-0103-x

**Published:** 2015-11-17

**Authors:** David Bellar, Nina R. LeBlanc, Brian Campbell

**Affiliations:** School of Kinesiology, University of Louisiana at Lafayette, Lafayette, LA 70503 USA

**Keywords:** Alpha glycerylphosphorylcholine, Strength, Human performance, Sport supplements

## Abstract

**Background:**

Ergogenic aides are widely used by fitness enthusiasts and athletes to increase performance. Alpha glycerylphosphorylcholine (A-GPC) has demonstrated some initial promise in changing explosive performance. The purpose of the present investigation was to determine if 6 days of supplementation with A-GPC would augment isometric force production compared to a placebo.

**Methods:**

Thirteen college-aged males (Means ± SD; Age: 21.9 ± 2.2 years, Height: 180.3 ± 7.7 cm, Weight: 87.6 ± 15.6 kg; VO_2_ max: 40.08 ± 7.23 ml O_2_*Kg^−1^*min^−1^, Body Fat: 17.5 ± 4.6 %) gave written informed consent to participate in the study. The study was a double blind, placebo controlled, cross-over design. The participants reported to the lab for an initial visit where they were familiarized with the isometric mid thigh pull in a custom squat cage on a force platform and upper body isometric test against a high frequency load cell, and baseline measurements were taken for both. The participant then consumed either 600 mg per day of A-GPC or placebo and at the end of 6 days performed isometric mid thigh pulls and an upper body isometric test. A one-week washout period was used before the participants’ baseline was re-measured and crossed over to the other treatment.

**Results:**

The A-GPC treatment resulted in significantly greater isometric mid thigh pull peak force change from baseline (t = 1.76, *p* = 0.044) compared with placebo (A-GPC: 98.8. ± 236.9 N vs Placebo: −39.0 ± 170.9 N). For the upper body test the A-GPC treatment trended towards greater change from baseline force production (A-GPC: 50.9 ± 167.2 N Placebo: −14.9 ± 114.9 N) but failed to obtain statistical significance (t = 1.16, *p* = 0.127).

**Conclusions:**

A-GPC is effective at increasing lower body force production after 6 days of supplementation. Sport performance coaches can consider adding A-GPC to the diet of speed and power athletes to enhance muscle performance.

## Background

Performance in sport is often determined by moments of extreme force production and power output [1]. While much of this can be attributed to muscular strength [2, 3], some adaptations to training can be neural in nature [4]. A study by Pensini, Martin and Maffiuletti [5] demonstrated that increases in torque associated with 4 weeks of eccentric exercise were likely the result of central (or neural) adaptation. Based upon current knowledge it appears that both central and peripheral adaptations are necessary to enhance performance in athletes. Therefore, it is important to study nutritional interventions that have the potential to augment either potential site of adaptation.

α Glycerylphosphorylcholine (A-GPC) is a substance that could potentially augment human performance by facilitiating neuro-muscular interaction. A-GPC has been shown to augment acetylcholine levels in neurons in rat CNS [6], and has been shown to maintain reaction time in humans following exhaustive exercise [7]. Additionally A-GPC is generally considered safe for consumption in moderate to high doses [8, 9]. Ingested A-GPC is converted to phosphatidylcholine, a source of choline [10]. Dietary choline levels are linked to the rate of biosynthesis of acetylcholine [11]. Given that cholinergic nerves trigger muscle contraction, and that choline availability is linked to acetylcholine synthesis substances that could augment choline availability might have the potential to influence muscular performance. To date some work has been done examining the ability of phospholipids to restore choline levels after exercise, but there is a dearth of information regarding the ability of compounds like A-GPC to acutely enhance performance [11]. The purpose of this study was to examine the effects of 6 days of supplementation with A-GPC on measures of isometric force production in the upper and lower body.

## Methods

**Fig 1:**
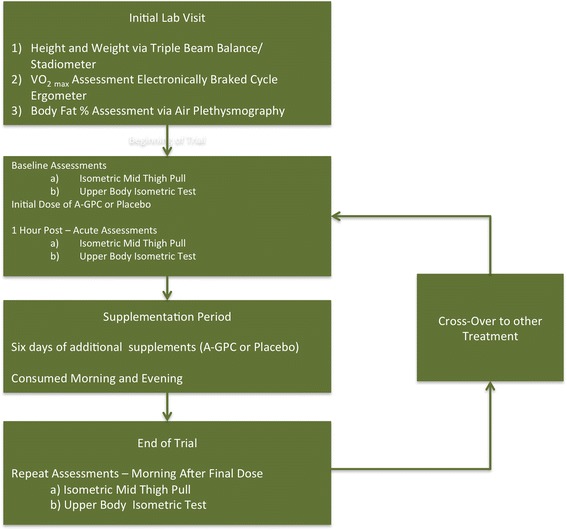
Flowchart of Experimental Procedures

The Institutional Review Board at the University of Louisiana at Lafayette reviewed the present investigation for ethics. The study was a double-blind, placebo-controlled crossover with a 1-week washout period that included 13 healthy, college-aged males (Means ± SD; Age: 21.9 ± 2.2 years, Height: 180.3 ± 7.7 cm, Weight: 87.6 ± 15.6 kg; VO_2_ max: 40.08 ± 7.23 ml O_2_*Kg^−1^*min^−1^, Body Fat: 17.5 ± 4.6 %). Subjects reported to the lab and give informed consent, which included consent to publish, prior to baseline assessments which included height and weight, an assessment of maximum aerobic capacity via a COSMED CPET system (COSMED, Rome ITL) with integrated electronically braked cycle ergometer as outlined in previous studies [12], and body fat percentage via air displacement plethysmography (Bod Pod Gold Standard System, COSMED Rome, ITL) . The following week trial one (random order: either placebo or 600 mg of A-GPC) began. For the trials baseline performance testing was done and they were given an initial dose (placebo or A-GPC) while in the lab, 1 h later the performance testing (isometric mid thigh pull, upperbody isometric test) was repeated. The subjects were then given 6 days of additional pre-packaged supplement to take (morning and evening). The subjects reported back on day 6 of this period to repeat performance testing after the final dose of supplement. After a 1-week washout period, the subjects repeated the trial with the other treatment. (see Fig. 1).

### Treatments

The treatments consisted of 600 mg daily of A-GPC (AlphaSize®, ChemiNutra, Austin, TX) or a placebo. Both treatments were administered in the same capsules (gel caps) and were the same color (white). The A-GPC capsules were supplied with a certificate of analysis from a third party lab confirming the amount of active ingredient. The placebo capsule consisted of microcrystalline cellulose and magnesium stearate (Nature’s Supplements, Carlsbad, CA USA). Both the participant and researcher were unaware of the identity of either treatment until the end of the study.

The participants were instructed to take doses in the morning and evening that would deliver a total of 600 mg of A-GPC per day and were given the pills in a non-distinct plastic bottle marked only with a code. The participants returned the bottles at the end of the study. The participants reported 100 % compliance with taking the required doses.

### Isometric mid thigh pull (IMTP)

The isometric mid-thigh pull test (IMTP) is a well-validated strength measure [13]. Testing was conducted in a customized power rack (Rogue Fitness, Columbus, USA) that is secured to a concrete laboratory floor surrounding a AMTI Force Plate (Advanced Materials Technologies Inc., Watertown USA). The power rack allows for small incremental adjustments in height for a steel bar that is secured via two large tubular steel members.

The participant was instructed to stand with the feet shoulder width apart above the force plate. The height of the bar was adjusted so that the participant was in a position where the torso was upright (assessed via a contractors box level), the knees achieved between 120–130° of flexion (measured via a goniometer) and the arms were straight while holding the bar. The participants were told to "drive straight up" and to pull as hard as they could against the chain until the force began to noticeably decline. The peak force was assessed at a sampling rate of 2000 Hz using an AMTI Force Plate. Subjects were familiarized with the IMTP during the initial lab visit. Measurements were taken in triplicate with a five-minute rest.

### Upper body isometric test (UBIST)

The participants were positioned on three elevated platforms with the chest directly suspended over a load cell anchored into the concrete floor of the lab (iLoad Pro, Loadstar Sensors, Fremont CA). The load cell had a capacity of greater than 5000 N and a listed accuracy of 0.25 % for the full scale of measurement. The participants were placed in a push-up style position, with the hands at 150 % of biacromial width, and the elbows at 90° of extension (measured via a goniometer). A thick, non-elastic strap was run over one shoulder and under the opposite shoulder and connected with metal rings to a chain that was tethered to the load cell.

The participants were instructed to keep their backs flat, and push with their hands maximally until told to stop by the researcher. Prior to data capture the load cell was tared to ensure the weight of the load cell and apparatus were accounted for. The researcher started data collection and verbally instructed the participant to “push as hard as possible”. The participants were verbally encouraged during data collection, which was terminated when the force production declined by 50 N from the peak value registered. The load cell was set to capture data at maximum rate (150Hz) and the data was exported and analyzed in JMP 11.0 (SAS Institute Inc, Cary NC). Peak force values were isolated from the data and used for subsequent analysis. The test was performed three times with 5 min rest between assessments. The validity and reliability of this test have been reported in the literature [14].

### Statistical analysis

Reliability was assessed for the isometric tests via Intra Class Correlation Coefficients (ICC). Repeated measures Ancovas were used to examine acute (baseline and 1 h post) and chronic (baseline and day 6) changes in performance between treatments. Order of administration (Placebo first, A-GPC first) was entered into the model as a covariate. G*Power software [15] was used to determine effect size (Cohen’s d), all other analyses were performed using a modern statistical software package (JMP, version 11.0 SAS Institute Inc., Cary, NC). Magnitude based inferences were calculated to assist with interpretation of results [16]. The use of magnitude based inference is an attempt to expand the interpretation of findings to include harmful, trivial and beneficial as interpretations, rather than just significant, non-significant [17]. This interpretations in not without controversy [18], as such the authors have chosen to include it alongside a more traditional statistical approach.

## Results

### Reliability of isometric tests

The isometric tests demonstrated reliability when the triplicate measurements were examined via ICC (range: 0.969–0.984). Measurements were not different at any time points (*p* > 0.05). Therefore in subsequent analysis the peak value from the set of three measures was used.

### Treatment effects—acute

Repeated measures Anova did not reveal any main effects (F = 0.003, *p* = 0.9584) nor interaction effects of treatment*time (F = 0.114, *p* = 0.738) for IMTP performance 1 h after the initial dose of A-GPC or Placebo. Similar results were revealed when UBIST performance was analyzed.

### Treatment effects—chronic

**Fig 2:**
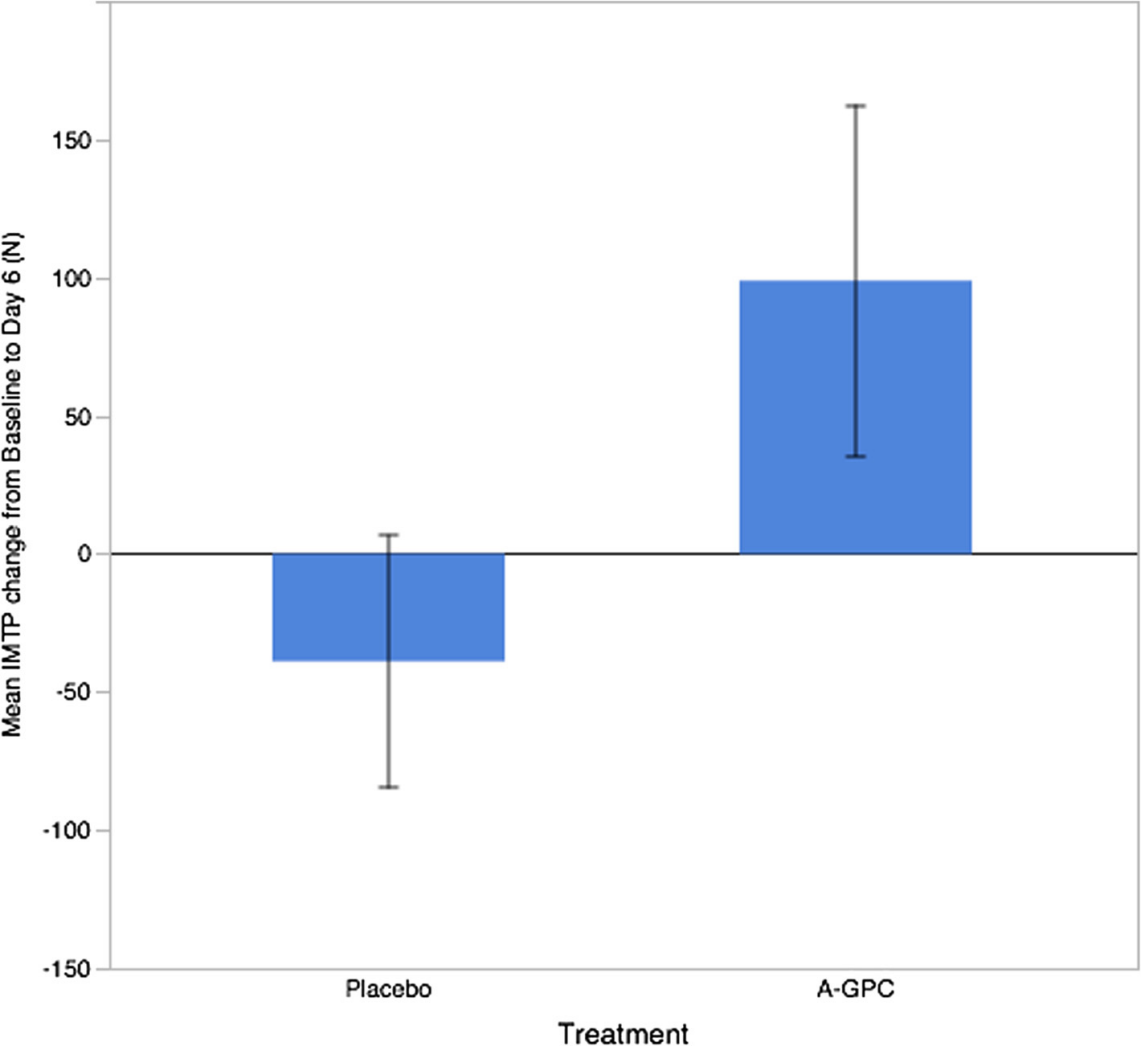
Mean change in Isometric Mid Thigh Pull Peak force after 6 days of supplementation with A-GPC. Error bars represent +/− 1 SEM

Repeated measures Anova revealed a significant interaction effect for treatment (A-GPC vs Placebo) by time (baseline, day 6) for IMTP peak performance (F = 3.12, *p* = 0.04; change from baseline A-GPC: 98.8. ± 236.9 N vs Placebo: −39.0 ± 170.9 N, ES = 0.961). See Fig. 2.

**Fig 3:**
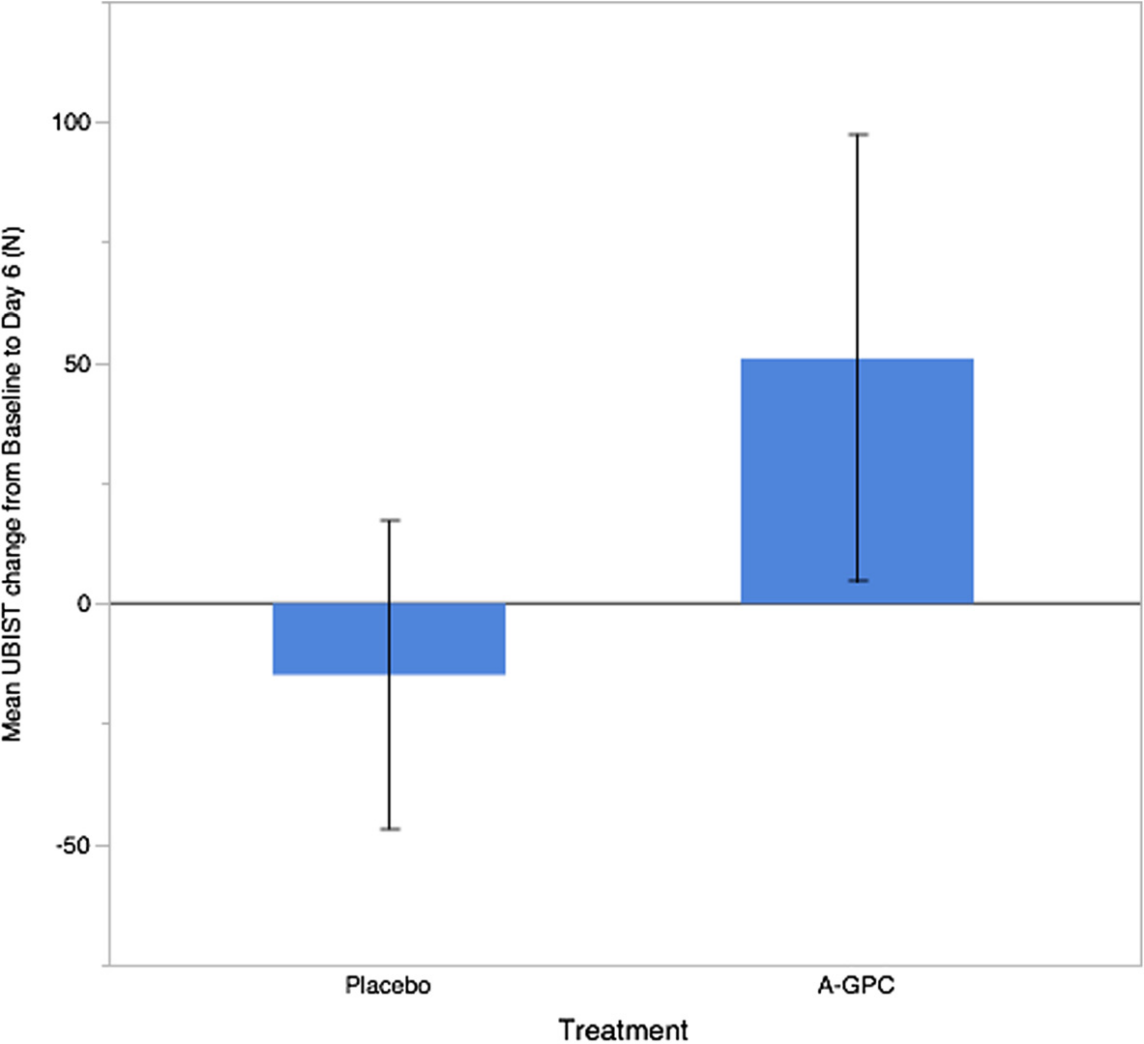
Mean change in Upperbody Isometric Test force after 6 days of supplementation with A-GPC. Error bars represent +/− 1 SEM

For the upper body test the A-GPC treatment trended towards greater change from baseline force production (A-GPC: 50.9 ± 167.2 N Placebo: −14.9 ± 114.9 N) but the interaction effect of treatment by time failed to obtain statistical significance (F = 1.36, *p* = 0.127). However, this data (see Fig. 3) demonstrated a large effect size (ES = 0.714). This suggests that the variability of the subject’s upper body strength limited the statistical power, however, it if likely that a real effect exists in this data. Magnitude based inferences suggest that the A-GPC was 68.3 % likely beneficial for increasing upper body isometric force and 86.5 % likely beneficial for increasing lower body isometric force production.

## Discussion

The results of this study support the use of A-GPC to enhance strength, particularly in the lower body after 6 days of administration of a 600 mg dose. The literature does not contain controlled experimental data regarding the effects of A-GPC on aspects of human performance directly related to isometric strength, and thus this study represents a first step in the evaluation of this product for such use. The literature does contain some evidence that choline itself is important to consider in regard to endurance performance [19, 20]. The current literature does contain some information about A-GPC and performance measurements. Jagim et al. [21] reported that a multi-ingredient supplement that contained A-GPC enhanced mean power during a maximal effort sprint test on a non-motorized treadmill but did not produce any changes in counter movement jumping performance peak or mean power. Parker et al. [22] reported acute supplementation with 200 mg or 400 mg of A-GPC did not statistically enhance performance, thought the authors did note a non-significant trend in vertical jump peak power. Acute supplementation with 600 mg of A-GPC has been shown to augment bench press power in a small sample of men with 2 years of training experience [23]. This study is similar in finding to the present investigation in dose of A-GPC administered (600 mg) and suggests enhancements in performance. These previously reported studies on A-GPC vary greatly in design, measurements and administrations. The lack of consistency of doses (200–600 mg) and time of administration (30–90 min prior to activity) may explain the lack of consistent findings. Given the present evidence in the literature, further studies will be needed to confirm the results reported from this experiment, the data represent a promising start and suggest alternative uses for A-GPC.

The potential mechanism by which A-GPC could confer enhanced strength and power performance involves increased bio-available choline, which may result in augmented acetylcholine synthesis in neurons. A-GPC has been shown to augment acetylcholine levels in CNS neurons [6]. Evidence suggests that when administered intramuscularly A-GPC can increase plasma choline levels [24]. A-GPC has also been shown to increase growth hormone secretion though the action of acetylcholine stimulated catecholamine release [25]. This increase in cholinergic tone and associated increased growth hormone release was also reported in old and young subjects after administration of growth hormone releasing hormone in conjunction with A-GPC [26]. In the present investigation it is unlikely a moderate increase in growth hormone over the course of 7 days would have impacted maximum strength although this evidence suggests that longer chronic studies of A-GPC may be warranted as chronic elevations in growth hormone could potentially further augment performance.

While the present study presents positive preliminary findings for A-GPC augmenting strength, it is not without limitation. The present investigation is limited by sample size. The study will need to be replicated with larger study populations and alternative measures of human performance, likely those that have the capacity to measure power not just peak force. Additionally, different does of A-GPC need to be explored to determine any potential dose-response, or lower limit for meaningful effect. We suggest that in vitro studies may also be warranted to demonstrate that A-GPC has the potential to augment neurotransmitter levels in motor neurons. These studies can help to clarify the timing of A-GPC administration, which may in turn result in studies with a more targeted and informed dosing scheme.

## Conclusions

The results of the study suggest that A-GPC is effective at increasing lower body force production after 6 days of supplementation. A similar trend was noted in upper body isometric strength, however; this failed to attain statistical significance. Given that in many sports it is understood that a very small change in performance, often times less than 2 %, can significantly affect outcomes it is important to note that the 6 days of A-GPC resulted in greater than a 3 % increase in lower body isometric strength. Sport performance coaches can consider adding A-GPC to the diet of speed and power athletes to potentially enhance muscle performance.

## Abbreviations

A-GPC: Alpha glycerylphosphrylcholine
IMTP: Isometric mid thigh pull
UBIST: Upper body isometric test
